# Evaluation of Performance and Tunability of a Co-Flow Inertial Microfluidic Device

**DOI:** 10.3390/mi11030287

**Published:** 2020-03-10

**Authors:** Amanda Bogseth, Jian Zhou, Ian Papautsky

**Affiliations:** 1 Department of Bioengineering, University of Illinois at Chicago, Chicago, IL 60607, USA; 2 University of Illinois Cancer Center, Chicago, IL 60612, USA

**Keywords:** microfluidics, particle separation

## Abstract

Microfluidics has gained a lot of attention for biological sample separation and purification methods over recent years. From many active and passive microfluidic techniques, inertial microfluidics offers a simple and efficient method to demonstrate various biological applications. One prevalent limitation of this method is its lack of tunability for different applications once the microfluidic devices are fabricated. In this work, we develop and characterize a co-flow inertial microfluidic device that is tunable in multiple ways for adaptation to different application requirements. In particular, flow rate, flow rate ratio and output resistance ratio are systematically evaluated for flexibility of the cutoff size of the device and modification of the separation performance post-fabrication. Typically, a mixture of single size particles is used to determine cutoff sizes for the outlets, yet this fails to provide accurate prediction for efficiency and purity for a more complex biological sample. Thus, we use particles with continuous size distribution (2–32 μm) for separation demonstration under conditions of various flow rates, flow rate ratios and resistance ratios. We also use A549 cancer cell line with continuous size distribution (12–27 μm) as an added demonstration. Our results indicate inertial microfluidic devices possess the tunability that offers multiple ways to improve device performance for adaptation to different applications even after the devices are prototyped.

## 1. Introduction

Microfluidic systems have emerged as viable alternatives to the conventional benchtop methods for separation and purification of cells [1,2,3]. Such systems are generally classified as either active, those relying on electric, magnetic or acoustic forces or passive, those relying on hydrodynamic forces. While active systems offer more accurate and selective manipulation of cells, their throughput is inherently low. Thus, passive systems, such as deterministic lateral displacement (DLD) [4,5], pinched flow fractionation (PFF) [6,7], hydrodynamic filtration [8,9] and inertial focusing [10,11,12], have gained popularity. In particular, inertial focusing is especially attractive due to simpler device designs since separation occurs by hydrodynamic forces only, while DLD and PFF require specific geometric designs [3]. Inertial focusing also has relatively high throughputs as compared with hydrodynamic filtration [9,13]. A number of recent reviews on inertial microfluidics have highlighted the underlying physical principles and have described the promising applications from enrichment of particles to medical diagnoses [3,14,15,16]. 

Development of microfluidic devices generally begins with validation and optimization using microparticles. This is especially true in inertial microfluidics, where microparticles have been and still are used to demonstrate new devices concepts, understand device performance and elucidate device physics [3,17,18,19,20,21,22,23,24,25,26,27,28,29]. These experiments often determine the separation efficiency and the resulting purity, allowing for optimization of flow conditions without wasting any of the biological sample. For biological applications, the size of the particles within the sample must be known so a device can be made to fractionate the sample into groups with specific cutoff sizes. Additionally, depending on the application, the samples may have a wide size range requiring multiple devices with various channel lengths. To avoid designing multiple channels, an inertial microfluidic device can be ‘tuned’ to sort the sample with a specific cutoff size using output channel resistance. Wang and Papautsky [30] demonstrated that output resistance and flow rate can be used to dynamically change the cutoff size of the outlets in a vortex separator. Tu et al. [10] demonstrated that output resistance changes the quality of concentrating a cell sample, as an alternative to centrifugation. These findings offer a simpler way to process diverse samples within the same device. 

**Figure 1:**
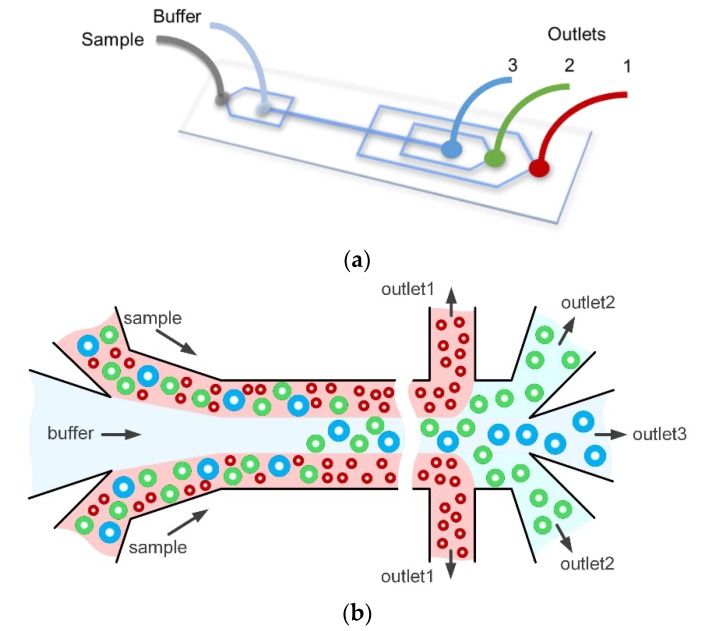
Illustrations of device setup and particle behaviors in device. (**a**) Schematic of device. The buffer enters in the first inlet and the sample behind, resulting in the sample nearest the walls and the buffer in the center. (**b**) Schematic of particle migration in an ideal situation with the smallest particles exiting in outlet 1, the largest in outlet 3 and those in between in outlet 2.

In this paper, we develop and characterize a co-flow inertial microfluidic device which shows tunability of separation by multiple parameters including flow rate, flow rate ratio and resistance ratio. Low-cost commercial microbeads of a continuous diameter range (2–32 µm) were separated into three outlets in the device (Figure 1a). The results show the possibility of separation refinement and flexibility of cutoff size. The results also suggest constraints for the refinement of a continuous range of particles because of the minimal size differences between particles near the cutoff size of an outlet. As shown previously [9,29], changing the resistance ratio of the outputs had the largest effect on flexibility of cutoff size, while flow rate and flow rate ratio had a smaller effect. Using these data, particles of sizes 7 μm, 15 μm and 26 μm were used to mimic biological sample and test the cutoff size requirements. An optimized separation is illustrated in Figure 1b, with the smallest particles exiting through outlet 1, the largest particles traveling to outlet 3, and those in between entering outlet 2. These experiments led to higher efficiency than the continuous range of particles because the large diameter difference between each particle led to specific equilibrium positions away from streamlines. These three-sized particle analyses validated the data demonstrated by the continuous range of particles, suggesting that the lower-cost alternative provides a better approximation of the device performance given that biological components, like cells, come in a large distribution of sizes. 

## 2. Experimental Methods 

### 2.1. Microfabrication

Microfluidic devices were fabricated in polydimethylsiloxane (PDMS) using the standard soft lithography process with dry photoresist masters, as we detailed previously [31]. Briefly, 3″ silicon wafers were dehydrated for 15 min on a 225 °C hotplate, laminated with a 50 μm thick ADEX film (DJ Microlaminates Inc., Sudbury, MA, USA) and baked for 5 min on a 65 °C hotplate. Next, the wafers were exposed to UV light (I-line 365 nm, Optical Associates Inc., San Jose, CA, USA) for 33 s at 10 mW/cm^2^ through a mask plate in hard contact. The wafers were developed in cyclohexanone (98%, Acros Organics, Pittsburg, PA, USA), washed with isopropyl alcohol (IPA) and deionized (DI) water, air dried and baked for 90 min on a 170 °C hotplate. A mixture of a 10:1 ratio of PDMS (Sylgard 184, Dow Corning, Midland, MI, USA) and curing agent was cast on the master, degassed for 90 min in a vacuum oven and cured for 120 min at 80 °C. Devices were cut out using a scalpel and inlet and outlet ports were cored using a biopsy punch with a diameter of 1.5 mm. Finally, devices were bonded to standard microscope glass slides using an oxygen plasma treatment at 10 W for 20 s (PE-50, Plasma Etch Inc., Carson City, NV, USA), baked for 60 min at 80 °C and allowed to cool to room temperature before use. 

### 2.2. Sample Preparation

For experiments involving non-fluorescent polymethyl methacrylate (PMMA) microparticles, a saline buffer was first prepared by mixing 2 g of NaCl (Thermo Fisher Scientific Inc., Waltham, MA, USA) with 10 mL of DI water. The PMMA microparticles (Cospheric LLC, Santa Barbara, CA, USA) with continuous size range 2–32 µm in diameter were then mixed with the prepared saline buffer at a concentration of ~3 million particles/mL (0.134 g in 50 mL of saline buffer). Buffer solution was used in preparing sample solution in order to match the particle density of 1.2 g/cm^3^ to achieve neutral buoyancy and minimize particle sedimentation in the syringe during experiments. 

For experiments involving fluorescent polystyrene microparticles, a saline buffer was first prepared by mixing 1.6 g of NaCl (Fisher Scientific Inc., Waltham, MA, USA) with 10 mL of DI water to match the particle’s density of 1.06 g/cm^3^. The polystyrene particles of diameter 7.32 µm and 15.45 µm (Bangs Laboratories Inc., Fishers, IN, USA), 18.67 µm and 26.3 µm (Polysciences Inc., Warrington, PA, USA) were mixed with the prepared saline buffer at a concentration of ~5 million particles/mL. Tween 80 was added at 0.1% *v*/*v* (Fisher Scientific, Waltham, MA, USA) to all particle solutions to minimize aggregation and avoid clogging.

### 2.3. Flow Experiments 

Syringes carrying sample and buffer solution was attached to the device using tubing of diameter 1.5 mm. Using syringe pumps, buffer solution was used to prime each channel before use. Particle solution was then added into the channel at an appropriate flow rate (150–750 μL·min^−1^) for ~2 min to allow stabilization of the flow before samples were collected. An inverted microscope (Olympus IX83 with Andor Zyla 5.5 camera, Andor Technology Ltd., Belfast, UK) was used to image samples in Bright Field and Fluorescence. A high-speed camera (FASTCAM Mini AX 200, Photron USA Inc., San Diego, CA, USA) at 1.05 µs exposure rate was used to capture bright-field images of the particles inside the microchannel. Images were compiled and analyzed using ImageJ®. Particle sizes of obtained samples were measured using software CellSens (Olympus Corp., Tokyo, Japan) for *n* = 300 particles. 

### 2.4. Cell Culture

Human non-small cell lung cancer cell line A549 was cultured in 25 cm^2^ flasks in completed Roswell Park Memorial Institute (RPMI) medium (Corning, Corning, NY, USA) containing 10% fetal bovine serum (FBS) (Gemini Bio, West Sacramento, CA, USA) and 1% antibiotic antimycotic solution (Sigma-Aldrich, St. Louis, MO, USA) in an incubator at 37 °C with 5% CO_2_. Cells were passaged once 70% confluency was reached to provide stable growing conditions. In preparation for experiments, the cells were extracted from flasks using 2 mL of 0.25% trypsin-ethylenediaminetetraacetic acid (EDTA) (Gibco, Waltham, MA, USA), incubated for 6 min and centrifuged for 5 min at 300 g. The cell pellet was re-suspended in 0.1% phosphate-buffered saline (PBS) at 100,000 cells/mL to be separated in the microfluidic device. After separation, cell sizes were measured using Cellsens software (Olympus Corp., Tokyo, Japan) and the viability of the cells were determined using Trypan Blue stain (ThermoFischer Scientific Inc., Waltham, MA, USA). After separation, cells were fixed using 80% ethanol for 30 min at −20 °C and stained using Hoechst 33342 (ThermoFisher Scientific Inc., Waltham, MA, USA) at a concentration of 1:300 for cell cycle analysis using flow cytometry conducted on the Gallios Flow Cytometer machine (Beckman Coulter, Brea, CA, USA). Gated data from the flow cytometer was analyzed using the Kaluza software (Beckman Coulter, Brea, CA, USA). 

## 3. Results and Discussion 

### 3.1. Inertial Focusing and Hydrodynamic Separation

As with other inertial microfluidic devices, the microfluidic chip we use in this work relies on inertial migration to achieve size-based separation of particles [13,32]. Thus, particles flowing downstream migrate across streamlines and order deterministically at equilibrium positions near channel walls. This ordering is caused by the balance of lift forces arising from the curvature of the velocity profile (the shear-induced lift *F_s_*) and the interaction between microparticles and the channel wall (the wall-induced lift *F_w_*). Under influence of these forces, microparticles rapidly equilibrate along each sidewall into bands where these two dominant lift forces balance each other. Once this initial equilibrium is reached, microparticle motion near channel sidewalls is dominated by the rotation-induced lift force *F_Ω_*, which drives them towards the center of channel sidewalls. In our co-flow microfluidic system, this means that particles can migrate out of the sample flow and into the central buffer flow. The size-selective aspect of the device arises from the inertial lift forces but more specifically from the rotation induced lift force (*F_Ω_* ∝ *a^3^*) in our low aspect ratio channel [32]. Consequently, the larger particles migrate across the streamlines faster than the smaller particles.

**Figure 2:**
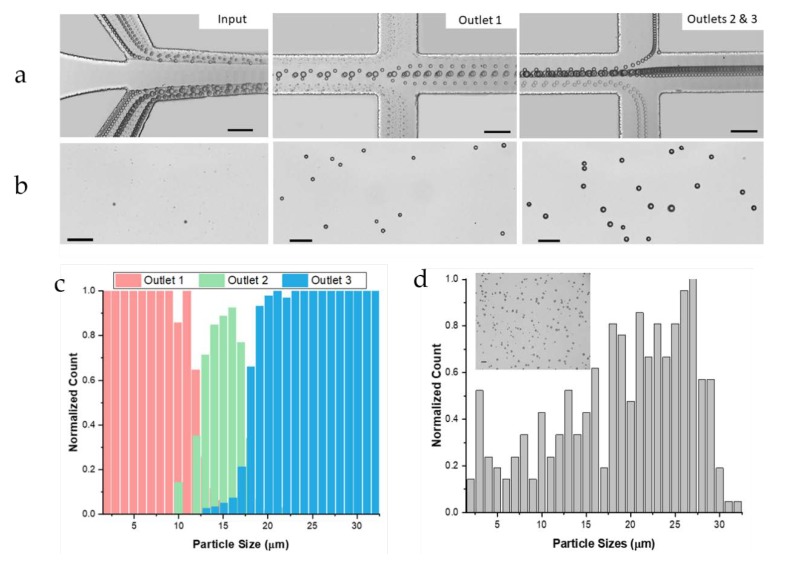
Separation of a continuous particle sample sized 2–32 μm in diameter at 300 μL·min^−1^, 1:4 flow ratio and 0.59 resistance ratio. Scale bars indicate 100 μm. (**a**) Bright field images at input and each outlet (1.05 μs exposure, stack *n* = 800). (**b**) Outlet samples. (**c**) The percentage of particles in each outlet for each size. (**d**) Initial particle sample sizes and bright field image.

Representative results illustrating device operation are presented in Figure 2. The randomly distributed particles in a sample of continuous size range 2–32 μm, are initially confined to the sidewalls. Under the influence of the shear-induced lift forces, as discussed above, the larger particles rapidly migrate to the center, while the smaller particles remain within the sample flow near sidewalls (Figure 2a). A symmetrical outlet system was used to remove the smallest particles, with diameter smaller than the set cutoff size (*a* < *a_c1_*), leaving the larger ones in the buffer flow. Further downstream, the largest particles which are focused near the channel centerline and have a larger diameter than the second cutoff size (*a* > *a_c2_*), exit through outlet 3. The mid-sized particles with diameters between the two cutoff sizes (*a_c1_* < *a* < *a_c2_*) exit through outlet 2 (Figure 2b). Histograms in Figure 2c confirm the cutoff sizes as *a_c1_* = 12 μm and *a_c2_* = 18 μm. The initial sample shows the visible size differences in the particles, with majority being a larger size (Figure 2d). More importantly, these results illustrate that particles with a size difference of ~2 μm can be separated with 70%–80% efficiency. With larger size difference of ~3 μm, separation efficiency is even higher, >90%. 

### 3.2. Analysis of Flow Rate

**Figure 3:**
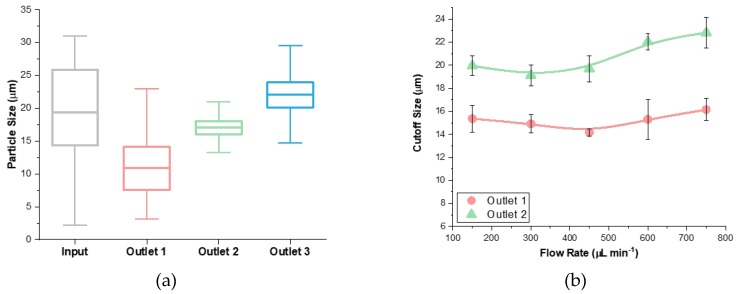

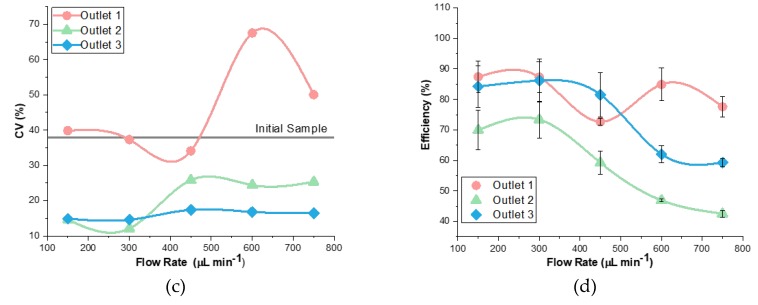
Sample flow rate impacts separation performance. (**a**) Fractionation of input sample into 3 outlets (300 μL·min^−1^, 1:2 ratio, 0.59 resistance ratio). The whiskers on the box plots show the full range of particle sizes. (**b**) Cutoff size stays constant in outlet 1 but increases in outlet 2 with flow rate. (**c**) Coefficient of variance is low at a low flow rate but increases with flow rate. (**d**) Efficiency of separation is high at a low flow rate but decreases rapidly beyond 300 μL·min^−1^.

Flow rate affects throughput and viability of cells post-separation [10,33]. Thus, flow rate optimization is a crucial step when developing a microfluidic device. Flow rates in the range 150–750 μL·min^−1^ (*Re* 40–200) were used to analyze the effect of flow speed on the flexibility of cutoff size and separation quality. For a high throughput system, flow rates lower than 150 μL·min^−1^ would not deliver sufficient throughput. Flow rates higher than 750 μL·min^−1^ would lead to deformation of PDMS causing disruptions in the microchannel. It has also been previously reported that efficiencies of separation at high flow rates are inadequate [34]. The separation quality was evaluated on the bases of the refinement of the 30 μm range sample fractionated within the outlets. Under the optimal 300 μL·min^−1^ flow rate, the device was able to decrease the distributions of the initial 30 μm size range to 20 µm, 17 µm and 21 µm size ranges at outlets 1, 2 and 3 respectively (Figure 3a). 

In general, increase of the total flow rate leads to the slight elevation of both cutoff sizes (Figure 3b). The cutoff size of outlet 1 changed only by ±1 µm. This negligible change is likely due to the inability of inertial forces to influence smaller particles within the limited downstream length of the microchannel. The cutoff size of outlet 2 increased from 19 µm to 23 µm as the flow rate increased, showing that the flow rate had an impact on larger particle’s focusing positions. This is mainly a time-limited case where increasing the flow rate resulted in less time for the particles to focus causing the larger particles to enter into outlet 2. This increased the average particle sizes and cutoff size of outlet 2. These results suggest that a microfluidic device’s cutoff sizes can only be changed with restriction to the size range of the particles.

The coefficient of variation (CV) of particles at each outlet was reduced at the optimal 300 μL min^−1^ flow rate (Figure 3c). Outlet 1 always had a higher CV than outlets 2 and 3 because the cutoff size was ~15 μm, which was the middle of the initial particle distribution. This reduced the ability to decrease the refinement of smaller particles because this device would always include half of the sample in outlet 1. Furthermore, the CV of outlet 1 was the same or higher than the initial sample for four of the data points. This is because the initial sample had a large standard deviation and a large average, while outlet 1 had a large standard deviation and a small average. Consequently, the CV does not give the correct impression when observing the refinement of the particles. The CV of the initial sample was smaller than outlet 1 but the distributions of the initial sample and outlet 1 were different size ranges. Therefore, the box plot is a better representation of refinement showing the outlet ranges and establishing the quality of device performance. However, the CV is important because it is able to compare the distributions of all three outlets relative to each other. 

The separation efficiency of the device decreased with increased flow rate in all three outlets (Figure 3d). This efficiency is defined as the percent fraction of target particles in their target outlets. At higher flow rates, particles have less time to achieve full focusing, effectively increasing the cutoff size (Figure 3b). Lower efficiency at higher flow rate is likely due to the increased sensitivity of performance on particle’s initial lateral positions. Since the lateral migration velocity scales with the square of downstream velocity (*U_L_* ∝ *U_f_^2^*) [32], slightly smaller particles initially located near the center might achieve focusing and exit into outlet 3, leading to the contamination of the outlet and thus decreased efficiency. Outlet 1 had the highest efficiency because it collected the smallest particles which were unable to cross the streamlines. The efficiency of outlet 2 was the lowest because the particle sizes overlapped with outlets 1 and 3. As a result, a lower flow rate resulted in better separation in terms of a high efficiency for all three outlets. 

### 3.3. Analysis of Flow Rate Ratio

**Figure 4:**
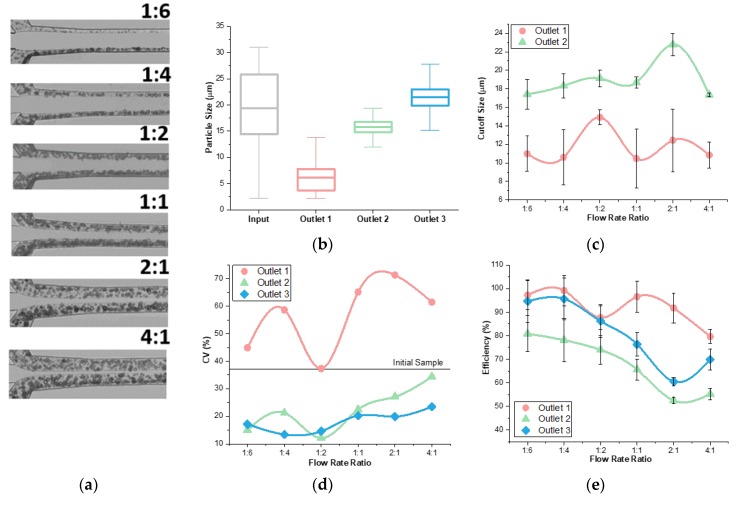
Influence of flow rate ratio on device performance. (**a**) Bright field images of flow show the boundary lines gets closer to the center and the width of the initial particle positions increases as the flow rate ratio increases. (**b**) The distribution of particles decreases from initial distribution in outlets for a flow rate of 300 μL·min^−1^, at a 1:4 ratio and a 0.59 resistance ratio. (**c**) Cutoff size in outlets 1 and 2 change negligibly when flow rate ratio increases. (**d**) Coefficient of variance increases with increasing flow rate ratio. (**e**) Efficiency of separation decreases when flow rate ratio increases.

**Figure A1:**
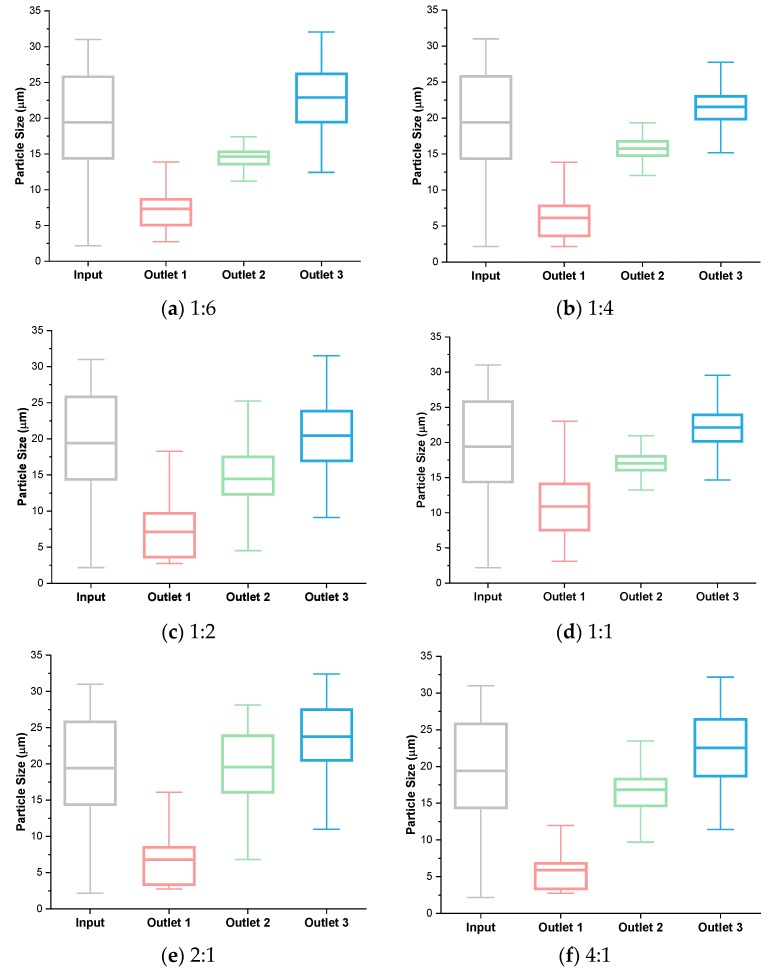
Distribution of particles within the outlets for various flow rate ratios. (**a**) 1:6 ratio had a larger distribution in outlet 3 compared to the 1:4 ratio. (**b**) 1:4 ratio had the most optimal refinement of the input distribution in all three outlets. (**c**) 1:2 ratio had a larger distribution in outlet 1 compared to the 1:4 ratio. (**d**) 1:1 ratio had a larger distribution in outlet 1 compared to the 1:4 ratio. (**e**) All outlets had larger distributions compared to the 1:4 ratio. (**f**) All outlets had larger distributions compared to the 1:4 ratio.

Flow rate ratio can impact the separation performance of a co-flow inertial system by controlling initial lateral positions of the suspended particles [20]. In this co-flow system, we defined flow rate ratio as the sample flow rate over the buffer flow rate. According to the size-dependent lateral migration and similar migration time in a given channel, a smaller ratio would be preferred as it gives similar initial lateral positions of particles regardless of size difference since all of them would be confined near sidewalls in narrow sample streams [20]. Six ratios were chosen to analyze the impact of flow rate ratio on separation quality and cutoff size flexibility (Figure 4a). The results confirm that a lower flow rate ratio produced better refinement of particles (Figure 4b). The comparison of all outlets can be found in Appendix A (Figure A1). As compared to the initial 30 μm range, outlets 1, 2 and 3 decreased to a 15 μm, 11 μm and 17 μm range, respectively. These decreased ranges showed better refinement at a 1:4 ratio compared to the 1:2 ratio above. While the 1:6 ratio also resulted in similar refinement and efficiency, the processing throughput is significantly lower. Therefore, the 1:4 ratio was used as the optimal condition for our co-flow channel. 

Our results further show the flow rate ratio slightly affects the cutoff sizes (Figure 4c). The trends that the cutoff sizes displayed were relevant to the conditions in the device. From the 1:4 to the 1:2 ratio, the cut off size increased for both outlets. The 1:2 ratio had a larger initial width for the sample, with random particle positions. As discussed above, the difference in particle initial lateral position warrants varying lateral migration distance even for particles with identical sizes. This caused some larger particles to go into outlet 1, increasing the cutoff size of the 1:2 ratio. Furthermore, as the flow rate ratio increased from the 2:1 to 4:1 ratio the cutoff size decreased. In a 4:1 ratio the sample initially filled up most of the channel (Figure 4a). With random initial positions of the particles, smaller particles were near the channel center more than when there was a smaller flow rate ratio. This caused smaller particles to enter into outlets 2 and 3 which decreased the cutoff size. Overall, our results suggest the influence of flow rate ratios on cutoff sizes of the device is small.

The CV indicated improved results with a lower flow rate ratio (Figure 4d). The trend shows an increase in CV as the flow rate ratio increased. Due to increased distributions at higher flow rate ratios, the efficiency showed a decreasing trend as flow rate ratio increased (Figure 4e). At a high flow rate ratio, the widely-distributed sample at the inlet yielded situations where particles near the channel wall did not have sufficient downstream length to migrate to their focusing positions, causing them to enter outlets prematurely. Additionally, some smaller particles would focus in the center and enter into outlets further down the channel. This caused a low efficiency because both small and large particles were entering into non-target outlets. Thus, these data suggest that a lower flow rate ratio results in better separation in terms of high efficiency of all three outlets.

### 3.4. Analysis of Resistance Ratio

**Figure 5:**
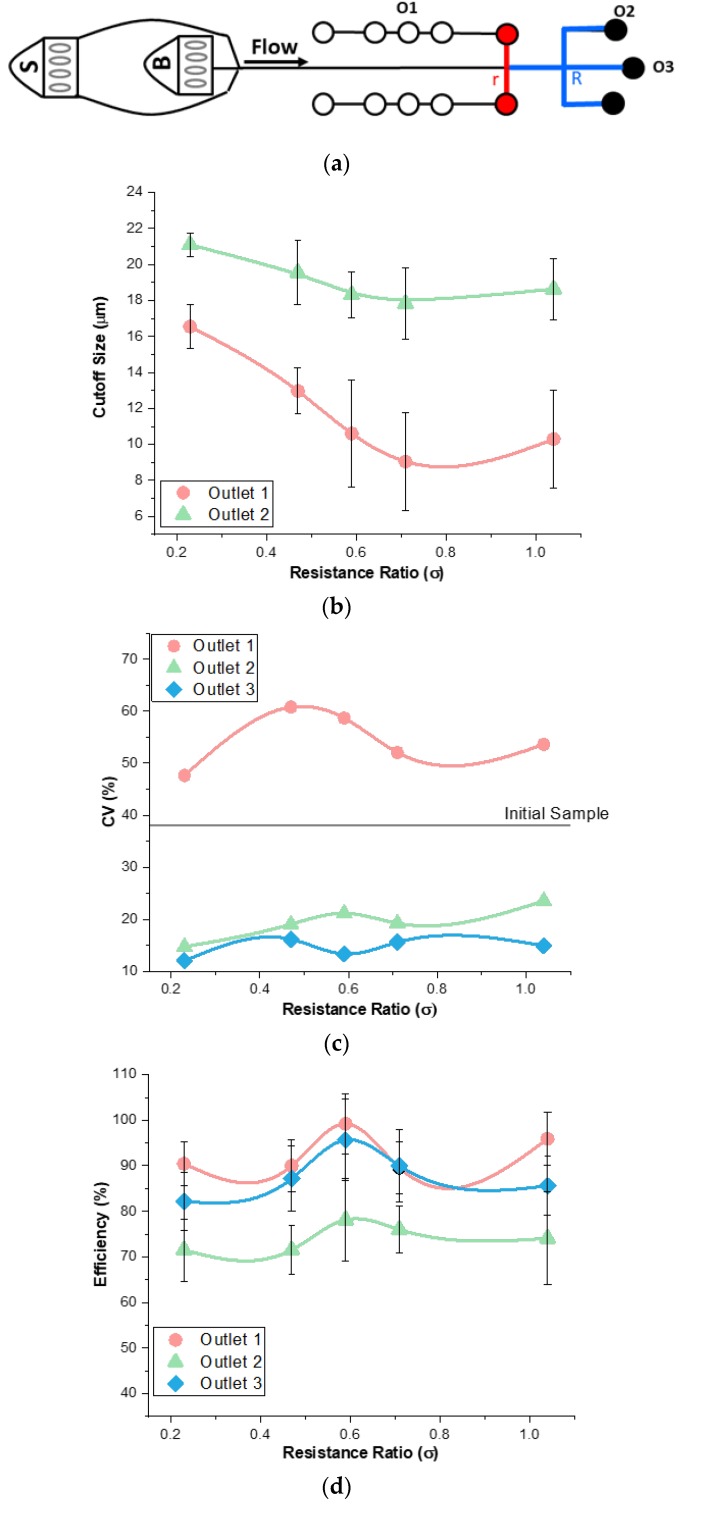
Influence of resistance ratio on device performance. (**a**) Schematic of resistance modification in outlet 1. (**b**) Cutoff size rapidly decreases in outlets 1 and 2 when resistance decreases. (**c**) Coefficient of variance (CV) decreases in outlet 1 and increases in outlets 2 and 3 as resistance ratio increases. (**d**) Efficiency of separation is highest when resistance is at σ = 0.59.

In addition to flow conditions, fluidic resistance of channels can be used to tune a microfluidic device to optimize separation. Fluidic resistance can be manipulated in multiple ways, including changing the external features of tubing and syringe pumps or internal features of aspect ratios or adding vortices in the channel [10,30,35,36]. In this work, we define the resistance ratio, σ = r/R, as the ratio of downstream lengths between outlets 1 and 2 (Figure 5a) [35]. We tested five different resistance ratios to assess the impact on cutoff size flexibility and separation quality. The results show that resistance ratio has a larger impact on cutoff size as compared to flow rate and flow rate ratio (Figure 5b). As σ increases, streamlines separating flows between side outlets and the main channel move closer to the sidewalls, resulting in a progressively smaller fraction of flow exiting through the side outlets. Since the particles equilibrate with the larger ones closer to the center and the smaller ones closer to the sidewalls, the shift in the separation streamline causes smaller diameter particles to enter outlet 1, resulting in a decreased cutoff diameter as a function of resistance ratio. The same occurs at outlet 2, with cutoff size also decreasing but by a smaller amount. In fact, the two outlets are coupled, with their cutoff sizes changing in tandem. These results illustrate that changing the external conditions offers more flexibility in tuning particle separation in the device. 

The concept of modifying the external channel resistance to tune separation quality is not entirely new, as it was previously demonstrated by us [29] and others [9]. It may be tempting to draw comparisons with our previous work on tunable vortex separators, where resistance ratio was also used for adjustments of the cutoff size. However, while the approach was the same, the results were different. This is because the vortex separators rely on inertial focusing, which leads the larger particles to focus closer to the sidewall and smaller particles closer to the channel centerline [29]. In this case, increasing resistance ratio between side and main channels yields an increase in cutoff size. Herein, the co-flow microfluidic system causes the larger particles to focus closer to the center and thus yields a decrease in the cutoff size as a function of resistance ratio.

The CV decreased as σ increased in outlet 1 and increased as σ increased for outlets 2 and 3 (Figure 5c). The trend of the cutoff size explains this. As σ increased, the cutoff size decreased which allowed a smaller range of particles to enter into outlets 1 and 2. Therefore, the particle sizes resulted in smaller standard deviations and averages. This resulted in lower ratios and a smaller CV. For outlets 2 and 3, the CV increased early on and then saturated at ±4%. Looking at outlet 2, the cutoff size decreased rapidly at first and then leveled off, so these curves match in describing the refinement of particles. These CV results did change significantly for outlet 1, suggesting that changing the resistance of the outlet did affect the refinement of separation.

The efficiency data suggests that a mid-σ is the best for the particles to focus efficiently. Insignificant changes occurred for efficiency trend except for the peak in the middle at σ = 0.59 (Figure 5d). In these experiments, the 1:4 flow rate ratio used gave each particle the same amount of length to focus. If the efficiency was entirely based on the particle’s lateral distance, then it would be the same for each σ. However, there was another mechanism that changed the efficiency which was the boundary line that allowed particles into the outlets. This boundary line only changes with alterations of the channel length. When σ was small, the boundary line moved closer to the channel center. This caused larger particles to enter into outlet 1. When σ was high, the boundary line moved closer to the channel wall. This caused only smaller particles to enter into outlet 1. When the resistance was in the middle of the channel, we hypothesize that the boundary line was at a point where the large particles have all focused and the small particles have not started focusing yet which caused the high efficiency. This is further evidenced in the cutoff size data explained above.

### 3.5. Separation of a Complex Particle Mixture 

**Figure 6:**
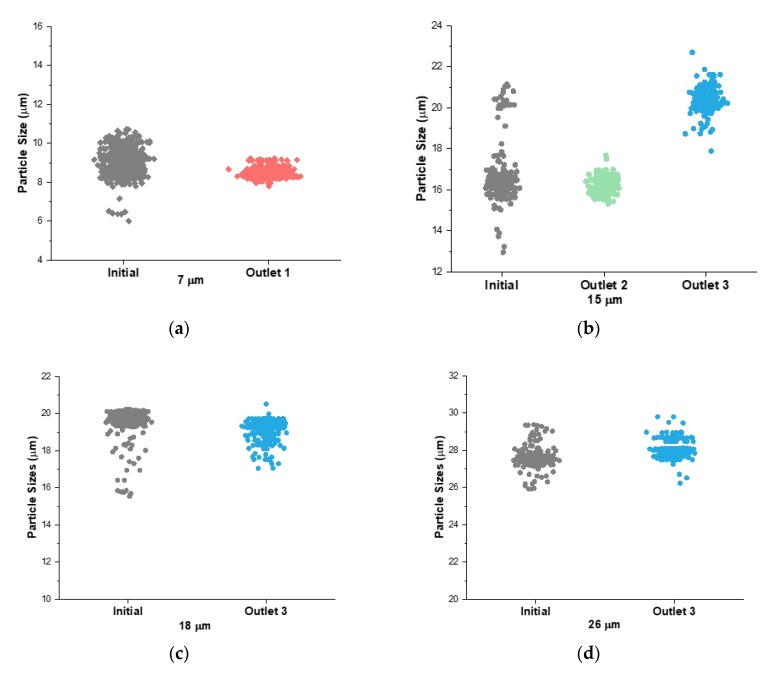
Single sized particles individually run through the device at 300 μL·min^−1^, 1:4 ratio, 0.59 resistance ratio. (**a**) All 7.32 μm particles went into outlet 1. (**b**) The 15.45 μm particles split between outlet 2 and outlet 3 with minimal overlap. (**c**) All 18.7 μm particles went into outlet 3. (**d**) All 26.3 μm particles went into outlet 3.

Following the determination of cutoff size flexibility, we evaluated the cutoff size of the device by flowing single sized particles individually through the channel to determine if they would separate as predicted. Using σ = 0.59 with a cutoff size *a_c1_* = 12 μm and *a_c2_* = 18 μm, we expected that the 7.32 μm particles would exit through outlet 1, the 15.45 μm particles would bimodally split into outlets 2 and 3 and the 18.67 μm and 26.3 μm particles would go into outlet 3. It is important to note the 15.45 μm particles had an initial size range of 15–22 μm and an average of 16.5 μm. Therefore, these particles would not exit in one outlet because their size range was in the middle of the cutoff size for outlet 2. The 7.32 μm particles were in the range 6–11 μm with an average of 9 μm. The 18.67 μm particles had a range of 16–21 μm and an average of 19.5 μm. The 26.3 μm particles had a range of 26–30 μm with an average of 27.5 μm. The blockage ratios for each particle were 14%, 30%, 37% and 52% for 7.32 μm, 15.45 μm, 18.67 μm and 26.3 μm, respectively. The experiments resulted with the 7.32 μm, 18.67 μm and 26.3 μm particles having an efficiency >95% (not shown) within their target outlets (Figure 6). The 15.45 μm particles split bimodally into outlets 2 and 3 with the cutoff size calculated as 18.32 μm, which agreed with the cutoff size calculated in Figure 4c (Figure 6b). 

**Figure 7:**
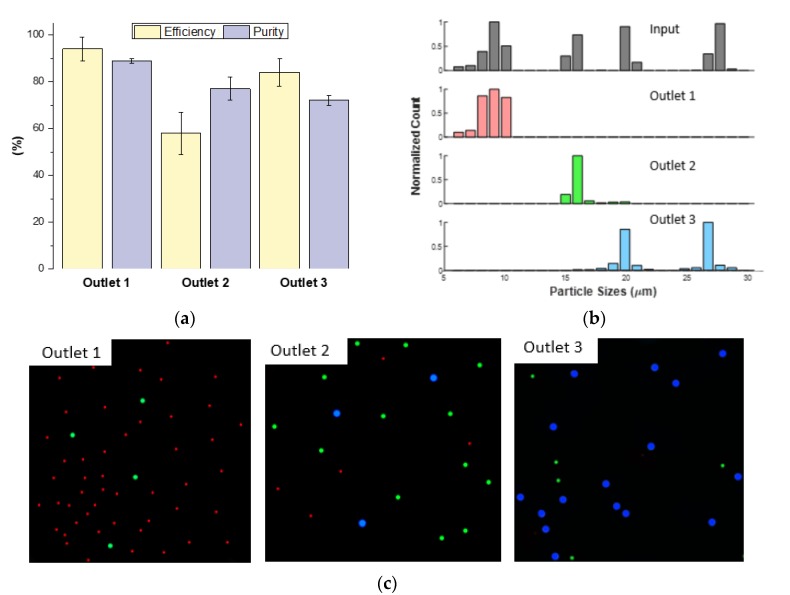
Mixed particle experiment of 7 μm (red), 15 μm (green) and 26 μm (blue) at 300 µL min^−1^, 1:4 ratio, 0.59 resistance ratio to show device optimization. (**a**) Purity and efficiency of outlets. (**b**) Initial particle sizes and particle sizes in outlets after experiments. (**c**) 10X images show the purity based on the amount of colors in each outlet.

We next challenged the device with a mixture of 7.32 μm, 15.45 μm and 26.3 μm diameter beads with the resistance ratio of σ = 0.59. Mixtures of two or three particle sizes are typically used to define cutoff sizes of a microfluidic device to prepare for biological sample [18,20,21,37]. Using a mixture also allowed for considerations of particle-on-particle interactions because of higher concentrations in the channel. For σ = 0.59, the results showed very high efficiency (>85%) for outlets 1 and 3 but as discussed above, the 15.45 μm beads yielded low purities for outlets 2 and 3 and consequently decreased the efficiency for outlet 2 (Figure 7a). 

### 3.6. Application to Cell Cycle Synchronization

**Figure 8:**
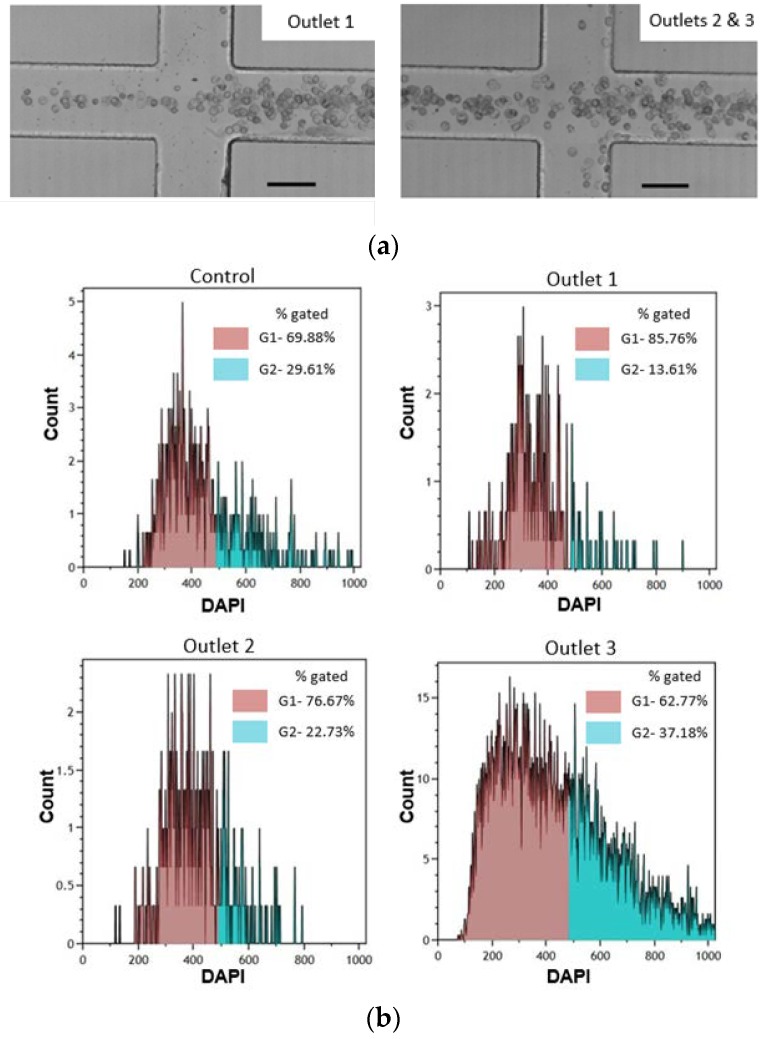
Representative results of the cell cycle synchronization. Flow conditions were 600 μL·min^−1^, 1:4 ratio and σ = 0.21. (**a**) Bright field images show cells exiting through outlets 1 and 2. Scale bar is 100 μm. (**b**) 4’,6-diamidino-2-phenylindole (DAPI) area analysis of cells shows increase of G1 phase in outlets 1 and 2 and an increase in G2 phase in outlet 3 as compared to the control.

The ability to study cells at a specific cell cycle phase is important for elucidating cellular mechanisms. Cell activity leading up to cell division can be characterized in four phases: gap phase G1, DNA synthesis phase S, gap phase G2 and mitosis phase M. Investigating the cell checkpoint at the G1 phase, which leads to cell proliferation or cell death, can allow for a better understanding of how a cell becomes cancerous. It has previously been shown that cell size is correlated to cell phase [38]. Thus, we used the microfluidic chip developed in this work to demonstrate enrichment of the G1 cell cycle phase of the A549 non-small cell lung cancer cell line. The A549 cells are approximately 12–27 μm in diameter and can be modeled with a continuous distribution of microparticles, as we have done earlier in this work. The chip was operated at 600 μL·min^−1^, 1:4 ratio and σ = 0.21. Figure 8a shows representative images of the microchannel with small cells exiting through outlet 1, larger cells exiting through outlet 2 and the largest cells exiting through outlet 3. The analysis of the three outlets using flow cytometry in Figure 8b clearly shows an enrichment of G1 phase from a 2.36 ratio in the control sample to 6.30 and 3.37 ratios for outlets 1 and 2, respectively. Here, the enrichment ratio is defined as percent fraction of gated G1/G2 cells. Outlet 3 collected both G1 and G2 phases, with an enrichment ratio of 1.68. The viability of cells post-separation was > 90%, which is comparable to post-separation viability reported by others [11,38], suggesting the microfluidic device can be used for separation of cells. In addition, the higher flow rate increased throughput, which is an added advantage of this method compared to the standard drug-induced methods for cell cycle synchronization [38,39].

## 4. Conclusions

In conclusion, we have comprehensively shown the impact of flow rate, flow rate ratio and output resistance on the tunability of cutoff size and the separation quality of a continuous range of microparticles in a co-flow device. Both flow rate and flow rate ratio offer convenient ways to fine tune the cutoff size of a given co-flow channel for separation. The former is more effective in fine tuning larger sized particles while better efficiency is achieved at lower flow rate. The latter is important for tuning separation quality as smaller ratio giving more uniform initial particle positions. The resistance ratio provides the most significant tunability in terms of cutoff size. Consequently, this also has a large effect on the refinement of separation due to the large flexible range of cutoff sizes. Overall, our results show that a lower flow rate, flow rate ratio and a mid-level resistance ratio provides the best separation efficiency and quality of all three outlets. Testing the device with a typical mixture of single sized particles validated the cutoff size showing that the continuous particles can be used to predict performance of the device.
